#  The Effect of Philadelphia and Pennsylvania Clean Indoor Air Act on Food Services and Drinking Places Sales and Numbers, 1998-2011

**DOI:** 10.5888/pcd10.130143

**Published:** 2013-11-27

**Authors:** Zhen-qiang Ma, Monica A. Fisher

**Affiliations:** Author Affiliations: Monica A. Fisher, Pennsylvania Department of Health, Harrisburg, Pennsylvania.

## Abstract

**Introduction:**

Philadelphia enacted its Clean Indoor Air Act (CIAA) nearly 2 years before the statewide CIAA. In this study, we assessed the economic impact of CIAAs on 4 types of food services and drinking places and addressed the predominant limitation of previous pre–post ban studies, namely the lack of control for confounders and changes in secular trends over time.

**Methods:**

We analyzed data from Pennsylvania Department of Revenue Quarterly 1998–2011 taxable county-level revenue sales and number of food services and drinking places. Region-specific and type-specific adjusted sales and number of food services and drinking places accounted for consumer spending as a general economic indicator. Segmented regression analysis of interrupted time-series methodology assessed changes in trend and level.

**Results:**

Pennsylvania CIAA had no significant effect on adjusted sales or numbers except for an increase in sales in Philadelphia for limited-service eating places and in the surrounding 4 counties for special food services. Philadelphia CIAA was associated with an increase in adjusted numbers of full-service restaurants in Philadelphia and the rest of the state, special food services in Philadelphia, and drinking places in the rest of the state, and a decrease in the number of special food services in the surrounding counties. Philadelphia CIAA had no significant effect on adjusted sales except for an increase in special food services in the rest of the state.

**Conclusion:**

Overall, CIAAs had no negative business-related impact and, for the most part, suggest a positive impact on restaurant sales and numbers. Our results provide further support for comprehensive CIAA ordinance for restaurants.

## Introduction

Pennsylvania has joined an ever growing number of places around the world that protect workers and the public from the health consequences of indoor secondhand smoke. The Pennsylvania Clear Indoor Air Act (CIAA) became effective in September 2008, 90 days after being signed into law in June 2008 (1). The Pennsylvania CIAA prohibits smoking in enclosed public places, including restaurants and drinking establishments that do not have legally approved exemptions. On January 8, 2007, before the enactment of Pennsylvania’s CIAA, Philadelphia County banned smoking in all indoor public places and workplaces, including restaurants and drinking establishments with similar exemptions (2). There are 2 types of drinking establishments (with or without a separate eating area) that can apply for exemptions from the CIAA if food accounts for 20% or less of overall sales and no one under the age of 18 is permitted to enter (1,2). The Philadelphia CIAA is the only local smoking ban in Pennsylvania; other local governments are preempted from regulating smoking more stringently than the state’s CIAA.

Even though numerous peer‐reviewed studies on the impact of smoke‐free policies on restaurant and drinking establishment revenues have found these policies to have no negative effect on sales (3–7), opponents of comprehensive CIAAs that do not permit exemptions continue to create concern regarding potential loss of revenue for these businesses due to a smoking ban. Opponents of the ban also disregard or question the evidence reported in many studies that smoke‐free policies may actually have a small but significant positive impact on sales (3,4,7–9). Reviews of studies assessing sales tax or business revenues, employment, and number of licensed establishments concluded that smoke‐free air legislation does not have an adverse economic impact on the hospitality industry (including restaurants, drinking establishments/bars) (7,8,10). In addition, a 2010 Cochrane qualitative narrative review (11) reported that smoking bans had no significant economic impact when measured as bar and restaurant attendance (12–15). Our study was designed to address the predominant limitation of pre–post ban studies identified in the Cochrane review, namely the lack of control for confounders and changes in secular trends over time (11).

The objective of this study was to assess the economic impact of CIAAs on food services and drinking places in Pennsylvania using a naturally occurring study designed to compare trends and level changes in the adjusted quarterly revenue sales and number of establishments. Because Philadelphia County enacted its CIAA almost 2 years before the rest of the state, we will compare food services and drinking places revenue sales in Philadelphia County versus the rest of the state, and in Philadelphia County versus the 4 large suburban Pennsylvania counties surrounding Philadelphia (Bucks, Chester, Delaware, and Montgomery). This approach addresses the proximity effect of the Philadelphia and Pennsylvania CIAAs and the predominant limitation of previous pre–post ban studies.

## Methods

Quarterly adjusted revenue sales and adjusted number of food services and drinking places were developed to take into account the general economic indicator of consumer spending. Food services and drinking places were defined as 4 distinct categories based on North American Industry Classification System (NAICS) codes: Full-Service Restaurants (NAICS code 7221), Limited-Service Eating Places (NAICS code 7222), Special Food Services (NAICS code 7223), and Drinking Places (alcoholic beverages) (NAICS code 7224). The overall quarterly taxable revenue sales and overall number of general merchandise stores (GMSs) (NAICS code 452) were used as a general economic indicator of consumer spending, similar to previous definitions using total retail sales (16).

Adjusted revenue sales were defined as the ratio of the sales for each type of establishment divided by the GMS sales:

1. Adjusted Full-Service Restaurants Sales = Full-Service Restaurants Sales/GMS Sales
2. Adjusted Limited-Service Eating Places Sales = Limited-Service Eating Places Sales/GMS Sales
3. Adjusted Special Food Services Sales = Special Food Services Sales/GMS
4. Adjusted Drinking Places (alcoholic beverages) Sales = Drinking Places Sales/GMS Sales

Adjusted Number of Food Services and Drinking Places were defined as the ratio of the number of type of establishment divided by the number of GMSs:

1. Adjusted Number of Full-Service Restaurants = Number of Full-Service Restaurants/Number of GMS
2. Adjusted Number of Limited-Service Eating Places = Number of Limited-Service Eating Places/Number of GMS
3. Adjusted Number of Special Food Services Places = Number of Special Food Services Places/Number of GMS
4. Adjusted Number of Drinking Places = Number of Drinking Places/Number of GMS

Regions in the state were categorized into 3 nonoverlapping regions: Philadelphia County, the 4 Pennsylvania counties surrounding Philadelphia (Bucks, Chester, Delaware, and Montgomery), and the rest of the state (61 counties, excluding 1 county because of an aberration of 99.9% loss in GMS). The time variable was defined as quarter (first through fourth) and year (1998–2011).

### Analytic approach

Segmented regression analyses of interrupted time-series methods (17) were used to assess the impact of both the Philadelphia and Pennsylvania CIAA on food services and drinking places. The analytic approach compared regions and changes in levels and trends of food services and drinking places’ adjusted revenue sales and adjusted numbers, following the 2 time points of the Philadelphia CIAA and Pennsylvania CIAA. These adjusted outcomes take into account the general economic indicator of consumer spending. This method controls for baseline level and trend when estimating expected changes due to the Philadelphia CIAA and the Pennsylvania CIAA. The following is the time-series regression equation for this analysis:


*Ŷ*_t_ = β_0_ + (β_1_ × Time) + (β_2i_ × CIAA_i_) + (β_3i_ × Time_post_CIAA_i_) + *Q*_rt_ + *e*_t_

*Ŷ*_t_ is the dependent outcome variable, with a separate model for each of the 8 dependent variables (adjusted full-service restaurant sales, adjusted limited-service eating places sales, adjusted special food services sales, adjusted drinking places sales, adjusted number of full-service restaurants, adjusted number of limited-service eating places, adjusted number of special food services, adjusted number of drinking places) for each of the 3 regions (Philadelphia, counties surrounding Philadelphia, and the rest of the state). Time is the number of quarters from the start of the observational period, starting from the first quarter of 1998 as 1, and then increasing by 1 for every quarter thereafter. CIAA is a dummy variable with a value of 0 for the segment before the CIAA and 1 for the segment at the beginning and post CIAA. Time_post_CIAA is the number of quarters at the beginning post CIAA with a value of 0 for the segment before the CIAA and 1 for the segment at the beginning of the CIAA that increases by 1 for every quarter thereafter. Subscript *i* equals 1 for the date of enactment of the Philadelphia CIAA and equals 2 for the date of enactment of the Pennsylvania CIAA. *Q*_rt_ is the quarter of the year for the sales and number of establishments and quarter 4 was the reference; *e*_t_ is the random variation at time t not explained by the model.

The intercept β_0_ coefficient estimates the baseline level of the adjusted sales/adjusted number of establishments at the beginning of the observation period. β_1_ estimates the trend for the adjusted sales/adjusted number of establishments before the enactment time of the CIAA. β_2_ estimates the change in adjusted sales/adjusted number of establishments level after the enactment of the CIAA measured as the change from the last time point before enactment of the CIAA to the first time point after the enactment of the CIAA. β_2_ can be considered to be an immediate change in the level of the adjusted sales/adjusted number of establishments due to the enactment of the CIAA. β_3_ estimates the change in the trend for the adjusted sales/adjusted number of establishments after enactment of the CIAA compared with the trend for the adjusted sales/adjusted number of establishments before the CIAA.

SAS Proc Autoreg (SAS Institute, Inc, Cary, North Carolina) was used for the analyses. The Durbin-Watson statistic was calculated to test for the serial autocorrelation of the error terms in the regression models. The Yule-Walker method with backstep option was used to correct autocorrelation (18). Initial auto-regressive parameters were set to 5 to account for quarterly changes. A quadratic term in time was incorporated in some models to reduce the autocorrelations in the model. Both positive and negative autocorrelations were nonsignificant in all final models. All final models had a Durbin-Watson statistic value close to the preferred value of 2. The statistical package SAS version 9.2 (SAS Institute, Inc, Cary, North Carolina) was used for all analyses. A *P* value of <.05 was considered significant.

## Results

### Full-service restaurants

**Figure 1:**
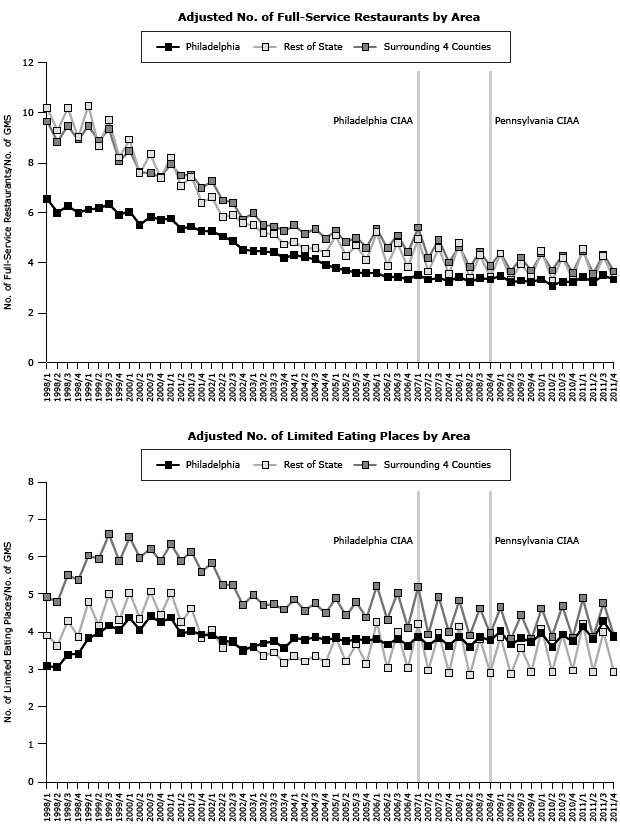

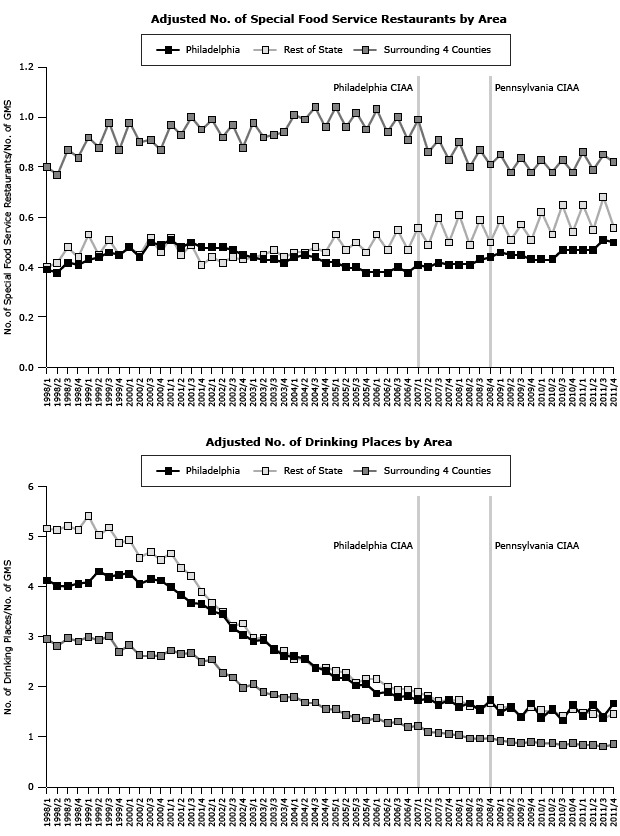
Effect of Philadelphia and Pennsylvania Clean Indoor Air Act (CIAA) on adjusted sales for each region and type of food service or drinking place, 1998–2011. Philadelphia County enacted a CIAA in January 2007, and the Pennsylvania CIAA became effective in September 2008; both dates are indicated by vertical lines.

**Figure 2:**
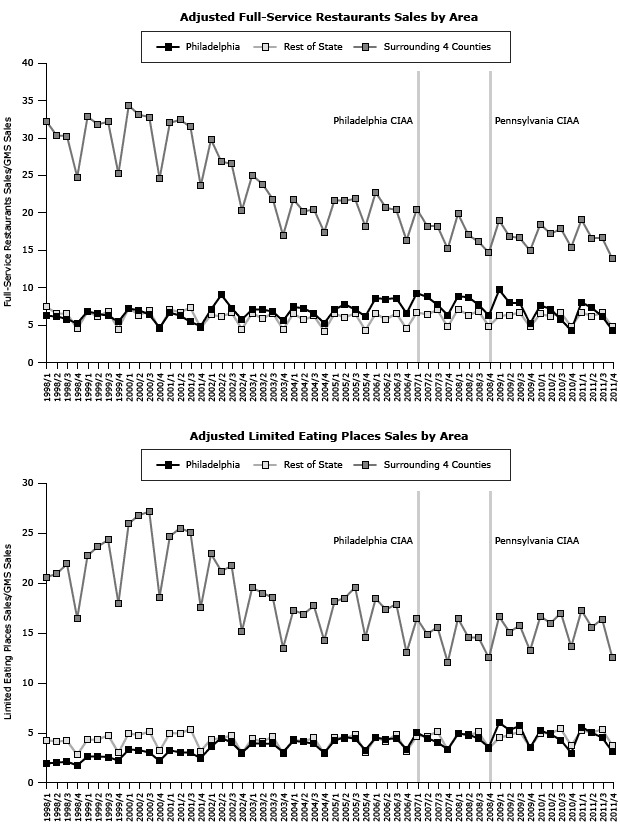

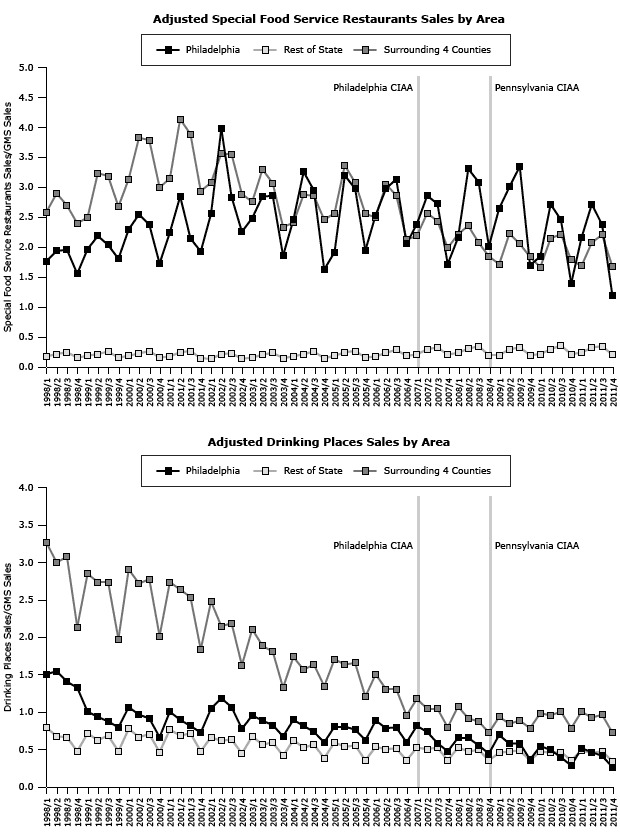
Effect of Philadelphia and Pennsylvania CIAA on adjusted number of types of food service or drinking place for each region, 1998–2011. Philadelphia County enacted a CIAA in January 2007, and the Pennsylvania CIAA became effective in September 2008; both dates are indicated by vertical lines.

Neither the Philadelphia CIAA nor the Pennsylvania CIAA had a significant impact on the trend or level of the largest component of the food services and drinking places market, namely, adjusted sales for full-service restaurant in any of the 3 regions (Philadelphia, surrounding counties, and the rest of the state) (Table 1, Figure 1). The trend for the adjusted number of full-service restaurants increased in Philadelphia (β_31_ = 0.079, *P* = .02) and the rest of the state (β_31_ = 0.191, *P* = .02) associated with Philadelphia’s CIAA. However, the Pennsylvania CIAA was not associated with any significant change in the trend or level of adjusted number of full-service restaurants (Table 2, Figure 2).

**Table 1:** Effect of Philadelphia and Pennsylvania CIAA on Adjusted Sales by Region and Type of Food Service or Drinking Place, 1998–2011

Type of Business	Philadelphia		Surrounding Counties		Rest of the State	
	β	*P* Value	β	*P* Value	β	*P* Value
**Full-service restaurant**	**Total R = 0.86**		**Total R = 0.98**		**Total R = 0.95**	
Intercept (β_0_)	4.275	<.001	29.217	<.001	4.890	<.001
Initial trend (β_1_)	0.051	<.001	−0.395	<.001	−0.017	.001
Philadelphia’s effect on rate (β_31_)	−0.154	.263	0.189	.584	0.038	.427
Pennsylvania’s effect on rate (β_32_)	−0.084	.572	0.094	.841	−0.015	.772
Philadelphia’s effect on level (β_21_)	0.995	.126	−0.435	.699	0.413	.077
Pennsylvania’s effect on level (β_22_)	0.323	.609	1.142	0.295	−0.274	.228
Quarter1	2.243	<.001	5.610	<.001	2.130	<.001
Quarter2	2.089	<.001	4.175	<.001	1.589	<.001
Quarter3	1.397	<.001	4.248	<.001	2.115	<.001
**Limited-service eating places**	**Total R = 0.91**		**Total R = 0.95**		**Total R = 0.96**	
Intercept (β_0_)	1.172	<.001	19.894	<.001	2.986	<.001
Initial trend (β_1_)	0.067	<.001	−0.195	<.001	0.003	.685
Philadelphia’s effect on rate (β_31_)	−0.076	.330	0.007	.982	0.030	.571
Pennsylvania’s effect on rate (β_32_)	−0.056	.506	0.186	.668	−0.001	.983
Philadelphia’s effect on level (β_21_)	−0.142	.700	−0.851	.411	0.051	.817
Pennsylvania’s effect on level (β_22_)	0.805	.031	1.232	.220	−0.028	.894
Quarter1	1.394	<.001	4.638	<.001	1.483	<.001
Quarter2	1.262	<.001	4.180	<.001	1.365	<.001
Quarter3	1.053	<.001	4.930	<.001	1.746	<.001
**Special food services**	**Total R = 0.79**		**Total R = 0.93**		**Total R = 0.96**	
Intercept (β_0_)	1.305	<.001	2.901	<.001	0.142	<.001
Initial trend (β_1_)	0.025	.001	−0.013	.161	0.001	.190
Philadelphia’s effect on rate (β_31_)	0.016	.799	−0.096	.128	0.006	.043
Pennsylvania’s effect on rate (β_32_)	−0.110	.115	0.095	.256	−0.004	.241
Philadelphia’s effect on level (β_21_)	−0.453	.133	−0.033	.873	0.021	.107
Pennsylvania’s effect on level (β_22_)	0.181	.533	0.128	.521	−0.030	.022
Quarter1	0.488	<.001	0.071	.302	0.022	.001
Quarter2	1.120	<.001	0.624	<.001	0.085	<.001
Quarter3	0.906	<.001	0.526	<.001	0.114	<.001
**Drinking places that serve alcohol**	**Total R = 0.92**		**Total R = 0.97**		**Total R = 0.96**	
Intercept (β_0_)	1.049	<.001	2.726	<.001	0.571	<.001
Initial trend (β_1_)	−0.014	<.001	−0.052	<.001	−0.006	<.001
Philadelphia’s effect on rate (β_31_)	−0.011	.664	0.020	.359	0.001	.875
Pennsylvania’s effect on rate (β_32_)	0.010	.752	0.036	.180	0.004	.539
Philadelphia’s effect on level (β_21_)	0.057	.614	−0.085	.376	0.005	.841
Pennsylvania’s effect on level (β_22_)	0.044	.672	0.015	.868	−0.002	.945
Quarter1	0.222	<.001	0.481	<.001	0.199	<.001
Quarter2	0.189	<.001	0.368	<.001	0.142	<.001
Quarter3	0.139	<.001	0.463	<.001	0.169	<.001

Abbreviation: CIAA, Clean Indoor Air Act.

**Table:** 

Year/Quarter	Full-Service Restaurant			Limited Service		
	Philadelphia	Rest of State	Surrounding 4 Counties	Philadelphia	Rest of State	Surrounding 4 Counties
1998/1	6.25	7.47	32.23	1.95	4.29	20.62
1998/2	6.19	6.55	30.33	2.04	4.15	20.93
1998/3	5.81	6.57	30.27	2.12	4.22	21.94
1998/4	5.25	4.51	24.78	1.77	2.81	16.48
1999/1	6.85	6.80	32.83	2.64	4.36	22.77
1999/2	6.52	6.19	31.84	2.66	4.33	23.66
1999/3	6.28	6.84	32.16	2.59	4.79	24.35
1999/4	5.47	4.49	25.23	2.25	3.03	17.99
2000/1	7.23	7.30	34.33	3.34	4.97	25.97
2000/2	7.00	6.37	33.11	3.29	4.79	26.83
2000/3	6.42	6.93	32.80	3.05	5.17	27.16
2000/4	4.67	4.51	24.58	2.21	3.21	18.62
2001/1	6.68	7.06	32.03	3.26	4.99	24.70
2001/2	6.29	6.76	32.43	3.07	4.96	25.52
2001/3	5.44	7.32	31.48	3.02	5.40	25.10
2001/4	4.89	4.66	23.61	2.49	3.20	17.55
2002/1	7.10	6.38	29.80	3.63	4.37	22.93
2002/2	9.06	6.18	26.84	4.50	4.48	21.22
2002/3	7.30	6.65	26.55	4.06	4.75	21.73
2002/4	5.76	4.50	20.34	3.01	3.00	15.17
2003/1	7.13	6.62	25.04	3.99	4.41	19.55
2003/2	7.09	5.88	23.85	3.92	4.13	19.02
2003/3	6.83	6.63	21.85	3.94	4.66	18.57
2003/4	5.58	4.42	16.98	3.09	2.96	13.50
2004/1	7.46	6.57	21.80	4.21	4.35	17.29
2004/2	7.25	5.71	20.20	4.11	4.13	16.92
2004/3	6.57	6.26	20.46	3.91	4.54	17.73
2004/4	5.28	4.22	17.43	3.02	2.92	14.26
2005/1	7.08	6.54	21.70	4.24	4.56	18.17
2005/2	7.80	6.06	21.63	4.60	4.46	18.51
2005/3	7.10	6.57	21.87	4.43	4.84	19.60
2005/4	6.19	4.32	18.16	3.30	3.01	14.60
2006/1	8.62	6.56	22.75	4.54	4.53	18.46
2006/2	8.50	5.76	20.67	4.38	4.20	17.36
2006/3	8.62	6.60	20.40	4.46	4.86	17.92
2006/4	6.59	4.54	16.34	3.36	3.16	13.06
2007/1	9.29	6.70	20.52	5.06	4.70	16.48
2007/2	8.78	6.45	18.25	4.48	4.69	14.90
2007/3	7.74	7.04	18.24	4.08	5.13	15.61
2007/4	6.34	4.77	15.20	3.36	3.31	12.09
2008/1	8.87	7.15	19.89	4.91	4.99	16.47
2008/2	8.74	6.37	17.07	4.87	4.73	14.59
2008/3	7.74	6.88	16.13	4.42	5.11	14.55
2008/4	6.29	4.84	14.70	3.59	3.48	12.52
2009/1	9.72	6.25	19.00	6.03	4.51	16.66
2009/2	8.02	6.36	16.85	5.30	4.81	15.10
2009/3	8.02	6.69	16.66	5.71	5.13	15.79
2009/4	5.20	4.78	14.96	3.51	3.58	13.25
2010/1	7.65	6.51	18.51	5.25	4.99	16.68
2010/2	7.17	6.19	17.19	4.87	4.97	16.01
2010/3	5.75	6.68	17.89	4.29	5.41	17.02
2010/4	4.31	4.83	15.42	3.00	3.72	13.63
2011/1	8.07	6.73	19.09	5.60	5.30	17.29
2011/2	7.32	6.23	16.61	5.03	5.02	15.59
2011/3	6.20	6.64	16.69	4.55	5.40	16.42
2011/4	4.35	4.77	13.87	3.13	3.75	12.53

**Table:** 

Year/Quarter	Special Food			Drinking Place		
	Philadelphia	Rest of State	Surrounding 4 Counties	Philadelphia	Rest of State	Surrounding 4 Counties
1998/1	1.77	0.18	2.59	1.51	0.80	3.27
1998/2	1.94	0.22	2.90	1.55	0.68	3.00
1998/3	1.96	0.25	2.70	1.41	0.67	3.08
1998/4	1.56	0.16	2.40	1.33	0.48	2.14
1999/1	1.97	0.19	2.50	1.01	0.72	2.85
1999/2	2.19	0.22	3.24	0.94	0.63	2.74
1999/3	2.04	0.26	3.19	0.88	0.69	2.74
1999/4	1.81	0.16	2.68	0.80	0.48	1.98
2000/1	2.30	0.19	3.14	1.07	0.78	2.91
2000/2	2.55	0.23	3.83	0.97	0.67	2.72
2000/3	2.38	0.27	3.79	0.92	0.71	2.78
2000/4	1.73	0.16	3.00	0.67	0.47	2.02
2001/1	2.25	0.18	3.15	1.01	0.77	2.74
2001/2	2.85	0.25	4.14	0.90	0.69	2.64
2001/3	2.15	0.27	3.89	0.82	0.72	2.54
2001/4	1.93	0.15	2.94	0.73	0.48	1.84
2002/1	2.56	0.14	3.08	1.05	0.67	2.48
2002/2	3.98	0.22	3.57	1.19	0.62	2.15
2002/3	2.84	0.23	3.55	1.07	0.64	2.19
2002/4	2.26	0.14	2.89	0.78	0.45	1.63
2003/1	2.49	0.16	2.76	0.96	0.68	2.11
2003/2	2.85	0.21	3.30	0.89	0.57	1.89
2003/3	2.86	0.24	3.07	0.83	0.60	1.81
2003/4	1.86	0.15	2.34	0.68	0.42	1.33
2004/1	2.46	0.18	2.41	0.90	0.63	1.74
2004/2	3.26	0.22	2.88	0.82	0.53	1.57
2004/3	2.95	0.26	2.86	0.74	0.57	1.64
2004/4	1.63	0.15	2.46	0.60	0.39	1.35
2005/1	1.92	0.19	2.57	0.81	0.60	1.71
2005/2	3.20	0.25	3.37	0.81	0.54	1.64
2005/3	2.98	0.27	3.08	0.77	0.56	1.66
2005/4	1.95	0.16	2.56	0.63	0.36	1.21
2006/1	2.54	0.18	2.50	0.89	0.54	1.51
2006/2	2.98	0.24	3.05	0.79	0.51	1.31
2006/3	3.14	0.29	2.87	0.80	0.52	1.31
2006/4	2.06	0.19	2.13	0.60	0.36	0.96
2007/1	2.39	0.21	2.19	0.82	0.53	1.18
2007/2	2.86	0.30	2.57	0.74	0.51	1.05
2007/3	2.73	0.33	2.44	0.59	0.53	1.05
2007/4	1.72	0.21	1.99	0.48	0.36	0.80
2008/1	2.16	0.24	2.22	0.66	0.53	1.08
2008/2	3.31	0.31	2.36	0.66	0.48	0.92
2008/3	3.09	0.35	2.08	0.56	0.50	0.88
2008/4	2.02	0.20	1.85	0.45	0.36	0.73
2009/1	2.65	0.20	1.72	0.70	0.46	0.94
2009/2	3.01	0.30	2.23	0.58	0.48	0.85
2009/3	3.35	0.33	2.06	0.59	0.49	0.89
2009/4	1.70	0.20	1.84	0.37	0.36	0.79
2010/1	1.85	0.22	1.67	0.55	0.48	0.99
2010/2	2.72	0.30	2.15	0.50	0.46	0.96
2010/3	2.46	0.36	2.22	0.40	0.47	1.01
2010/4	1.40	0.22	1.79	0.29	0.36	0.79
2011/1	2.16	0.24	1.69	0.52	0.49	1.01
2011/2	2.72	0.33	2.08	0.47	0.46	0.93
2011/3	2.38	0.35	2.21	0.42	0.48	0.97
2011/4	1.20	0.21	1.68	0.27	0.35	0.73

**Table 2:** Effect of Philadelphia and Pennsylvania CIAA on Adjusted Number of Specific Types of Food Service or Drinking Place by Region, 1998–2011

Type of Business	Philadelphia		Surrounding Counties		Rest of the State	
	β	*P* Value	β	*P* Value	β	*P* Value
**Full-service restaurants**	**Total *R* = 0.99**		**Total *R* = 0.99**		**Total *R* = 0.99**	
Intercept (β_0_)	6.624	<.001	9.164	<.001	9.313	<.001
Initial trend (β_1_)	−0.095	<.001	−0.148	<.001	−0.170	<.001
Philadelphia’s effect on rate (β_31_)	0.079	.021	0.105	.120	0.191	.024
Pennsylvania’s effect on rate (β_32_)	0.017	.671	0.019	.833	−0.031	.774
Philadelphia’s effect on level (β_21_)	0.149	.321	0.344	.166	0.092	.715
Pennsylvania’s effect on level (β_22_)	0.024	.865	0.044	.848	0.073	.763
Quarter1	0.131	.005	0.637	<.001	0.925	<.001
Quarter2	−0.035	.382	0.015	.766	−0.044	.391
Quarter3	0.115	.011	0.505	<.001	0.723	<.001
**Limited-service eating places**	**Total *R* a = 0.83**		**Total *R* a = 0.95**		**Total *R* = 0.92**	
Intercept (β_0_)	3.288	<.001	5.052	<.001	4.026	<.001
Initial trend (β_1_)	0.061	.013	0.050	.165	−0.028	.002
Philadelphia’s effect on rate (β_31_)	0.079	.199	0.123	.189	0.019	.757
Pennsylvania’s effect on rate (β_32_)	0.023	.692	0.051	.566	0.009	.915
Philadelphia’s effect on level (β_21_)	0.066	.661	0.187	.421	0.099	.654
Pennsylvania’s effect on level (β_22_)	0.077	.587	0.081	.713	0.044	.836
Quarter1	0.192	<.001	0.642	<.001	0.790	<.001
Quarter2	−0.015	.695	0.024	.664	−0.011	.829
Quarter3	0.172	<.001	0.567	<.0001	0.659	<.001
**Special food services**	**Total *R* a = 0.89**		**Total *R* a = 0.93**		**Total *R* = 0.87**	
Intercept (β_0_)	0.393	<.001	0.779	<.001	0.405	<.001
Initial trend (β_1_)	0.009	<.001	0.012	<.001	0.002	.021
Philadelphia’s effect on rate (β_31_)	0.017	.002	−0.018	.008	0.003	.539
Pennsylvania’s effect on rate (β_32_)	0.006	.205	0.030	<.001	0.001	.855
Philadelphia’s effect on level (β_21_)	0.026	.153	0.006	.851	0.015	.549
Pennsylvania’s effect on level (β_22_)	0.010	.556	−0.008	.767	−0.009	.700
Quarter1	0.007	.164	0.072	<.001	0.062	.001
Quarter2	−0.003	.582	0.005	.460	0.004	.549
Quarter3	0.014	.006	0.061	<.001	0.065	<.001
**Drinking places that serve alcohol**	**Total *R* = 0.99**		**Total *R* a = 0.99**		**Total *R* = 0.99**	
Intercept (β_0_)	4.697	<.001	3.067	<.001	5.555	<.001
Initial trend (β_1_)	−0.079	<.001	−0.035	<.001	−0.107	<.001
Philadelphia’s effect on rate (β_31_)	0.040	.261	0.059	.024	0.094	.009
Pennsylvania’s effect on rate (β_32_)	0.035	.465	0.022	.377	−0.005	.918
Philadelphia’s effect on level (β_21_)	0.011	.924	0.037	.650	−0.016	.894
Pennsylvania’s effect on level (β_22_)	0.040	.712	0.005	.946	0.053	.627
Quarter1	−0.091	.041	0.061	.040	0.019	.660
Quarter2	−0.010	.733	−0.003	.885	−0.024	.263
Quarter3	−0.108	.015	0.032	.267	−0.057	.193

Abbreviation: CIAA, Clean Indoor Air Act.

a Model includes quadratic term for time.

**Table:** 

Year/Quarter	Full-Service Restaurants			Limited Service		
	Philadelphia	Rest of State	Surrounding 4 Counties	Philadelphia	Rest of State	Surrounding 4 Counties
1998/1	6.55	10.19	9.65	3.09	3.90	4.92
1998/2	6.02	9.28	8.84	3.05	3.64	4.80
1998/3	6.27	10.19	9.48	3.40	4.29	5.52
1998/4	6.01	9.01	8.91	3.41	3.88	5.37
1999/1	6.13	10.29	9.47	3.83	4.80	6.03
1999/2	6.18	8.65	8.88	3.96	4.17	5.94
1999/3	6.34	9.69	9.34	4.17	5.01	6.60
1999/4	5.93	8.23	8.06	4.05	4.31	5.88
2000/1	6.06	8.91	8.49	4.37	5.05	6.51
2000/2	5.52	7.57	7.63	4.05	4.35	5.98
2000/3	5.84	8.35	7.58	4.41	5.07	6.22
2000/4	5.73	7.41	7.44	4.26	4.43	5.88
2001/1	5.76	8.22	7.94	4.37	5.05	6.34
2001/2	5.38	7.07	7.47	3.95	4.26	5.90
2001/3	5.44	7.46	7.54	4.02	4.62	6.14
2001/4	5.27	6.42	6.99	3.92	3.85	5.60
2002/1	5.26	6.61	7.27	3.91	4.06	5.84
2002/2	5.07	5.83	6.51	3.79	3.56	5.24
2002/3	4.87	5.92	6.41	3.71	3.76	5.26
2002/4	4.53	5.58	5.75	3.52	3.49	4.71
2003/1	4.48	5.50	5.99	3.61	3.59	4.99
2003/2	4.46	5.17	5.49	3.69	3.36	4.70
2003/3	4.41	5.16	5.48	3.76	3.46	4.74
2003/4	4.20	4.75	5.27	3.56	3.17	4.58
2004/1	4.32	4.84	5.49	3.84	3.35	4.87
2004/2	4.23	4.56	5.15	3.79	3.20	4.57
2004/3	4.14	4.60	5.37	3.86	3.37	4.76
2004/4	3.92	4.38	4.98	3.79	3.17	4.49
2005/1	3.80	5.08	5.27	3.83	3.86	4.89
2005/2	3.70	4.29	4.81	3.74	3.20	4.43
2005/3	3.61	4.67	5.03	3.81	3.67	4.79
2005/4	3.59	4.12	4.62	3.77	3.14	4.37
2006/1	3.59	5.23	5.36	3.81	4.27	5.23
2006/2	3.42	3.90	4.58	3.66	3.03	4.33
2006/3	3.45	4.79	5.12	3.82	3.99	5.04
2006/4	3.34	3.82	4.40	3.64	3.03	4.12
2007/1	3.53	4.95	5.40	3.88	4.19	5.20
2007/2	3.32	3.66	4.19	3.64	2.98	3.94
2007/3	3.40	4.60	4.93	3.84	3.96	4.93
2007/4	3.27	3.55	4.02	3.64	2.91	3.98
2008/1	3.45	4.79	4.66	3.88	4.14	4.84
2008/2	3.24	3.38	3.82	3.60	2.84	3.90
2008/3	3.39	4.31	4.44	3.87	3.82	4.63
2008/4	3.34	3.43	3.88	3.77	2.91	3.95
2009/1	3.48	4.36	4.39	4.01	3.88	4.64
2009/2	3.23	3.33	3.67	3.65	2.87	3.80
2009/3	3.29	3.96	4.18	3.83	3.58	4.45
2009/4	3.24	3.42	3.69	3.73	2.94	3.81
2010/1	3.35	4.46	4.36	3.96	4.07	4.63
2010/2	3.09	3.30	3.71	3.60	2.93	3.88
2010/3	3.26	4.21	4.28	3.92	3.94	4.69
2010/4	3.23	3.35	3.61	3.74	2.98	3.85
2011/1	3.44	4.55	4.46	4.13	4.20	4.88
2011/2	3.23	3.29	3.57	3.82	2.95	3.87
2011/3	3.50	4.27	4.35	4.28	3.99	4.77

**Table:** 

Year/Quarter	Special Food Services			Drinking Places		
	Philadelphia	Rest of State	Surrounding 4 Counties	Philadelphia	Rest of State	Surrounding 4 Counties
1998/1	0.39	0.40	0.80	4.13	5.15	2.95
1998/2	0.38	0.42	0.77	4.01	5.13	2.81
1998/3	0.42	0.48	0.87	4.00	5.21	2.96
1998/4	0.41	0.44	0.84	4.06	5.13	2.91
1999/1	0.43	0.53	0.92	4.08	5.41	2.99
1999/2	0.44	0.45	0.88	4.31	5.02	2.92
1999/3	0.46	0.51	0.98	4.19	5.17	3.01
1999/4	0.45	0.45	0.87	4.24	4.87	2.70
2000/1	0.48	0.48	0.98	4.26	4.93	2.83
2000/2	0.44	0.45	0.90	4.05	4.58	2.62
2000/3	0.50	0.52	0.91	4.14	4.69	2.62
2000/4	0.49	0.46	0.87	4.12	4.53	2.61
2001/1	0.51	0.52	0.97	3.98	4.67	2.73
2001/2	0.48	0.45	0.93	3.83	4.37	2.65
2001/3	0.50	0.49	1.00	3.67	4.22	2.67
2001/4	0.48	0.41	0.95	3.65	3.90	2.50
2002/1	0.48	0.44	0.99	3.51	3.67	2.53
2002/2	0.48	0.42	0.92	3.44	3.49	2.28
2002/3	0.47	0.44	0.97	3.17	3.21	2.19
2002/4	0.45	0.43	0.88	3.04	3.26	1.98
2003/1	0.44	0.44	0.98	2.91	2.96	2.04
2003/2	0.43	0.45	0.92	2.93	2.97	1.88
2003/3	0.43	0.47	0.93	2.75	2.72	1.83
2003/4	0.42	0.44	0.94	2.61	2.72	1.78
2004/1	0.44	0.46	1.01	2.60	2.55	1.80
2004/2	0.45	0.46	0.99	2.56	2.54	1.68
2004/3	0.44	0.48	1.04	2.39	2.36	1.68
2004/4	0.42	0.46	0.96	2.32	2.39	1.55
2005/1	0.42	0.53	1.04	2.17	2.31	1.54
2005/2	0.40	0.47	0.96	2.17	2.28	1.44
2005/3	0.40	0.50	1.02	2.02	2.07	1.37
2005/4	0.38	0.46	0.95	2.04	2.15	1.32
2006/1	0.38	0.53	1.03	1.86	2.15	1.37
2006/2	0.38	0.47	0.94	1.89	2.00	1.27
2006/3	0.40	0.55	1.00	1.79	1.94	1.29
2006/4	0.38	0.47	0.91	1.81	1.94	1.18
2007/1	0.41	0.56	0.99	1.72	1.88	1.22
2007/2	0.40	0.49	0.86	1.76	1.81	1.09
2007/3	0.42	0.60	0.91	1.64	1.71	1.08
2007/4	0.41	0.50	0.83	1.73	1.72	1.05
2008/1	0.41	0.61	0.90	1.59	1.73	1.02
2008/2	0.41	0.49	0.80	1.67	1.61	0.97
2008/3	0.43	0.59	0.87	1.52	1.55	0.95
2008/4	0.44	0.50	0.81	1.72	1.65	0.97
2009/1	0.46	0.59	0.85	1.48	1.57	0.92
2009/2	0.45	0.51	0.78	1.60	1.57	0.89
2009/3	0.45	0.57	0.84	1.39	1.40	0.86
2009/4	0.43	0.51	0.78	1.65	1.59	0.90
2010/1	0.43	0.62	0.83	1.37	1.52	0.86
2010/2	0.43	0.53	0.78	1.55	1.52	0.87
2010/3	0.47	0.65	0.83	1.32	1.41	0.83
2010/4	0.47	0.54	0.78	1.63	1.54	0.86
2011/1	0.47	0.65	0.86	1.41	1.49	0.83
2011/2	0.47	0.55	0.79	1.63	1.46	0.83
2011/3	0.51	0.68	0.85	1.40	1.36	0.80
2011/4	0.50	0.56	0.82	1.67	1.46	0.84

### Limited-service eating places

The only significant effect of the CIAAs was the association of the Pennsylvania CIAA with a positive immediate increase in Philadelphia’s adjusted sales for limited-service eating places (β_22_ = 0.805, *P* = .03) but not the trend (β_32_) (Table 1, Figure 1).

### Special food service restaurants

Neither the Philadelphia CIAA nor the Pennsylvania CIAA had a significant impact on the trend or level of the adjusted revenue sales for special food services in Philadelphia or the 4 surrounding counties. The Philadelphia CIAA was associated with an increase in the trend (β_31_ = 0.006, *P* = .04) for the adjusted special food services sales in the rest of the state but had no immediate impact (β_21_). The Pennsylvania CIAA was associated with an immediate decrease in adjusted special food services sales (β_22_ = −0.030, *P* = .02) in the rest of the state (Table 1, Figure 1), along with an increasing trend for the number of special food services restaurants in the 4 surrounding counties (β_32_ = 0.030, *P* < .001)( Table 2, Figure 2). The Philadelphia CIAA was associated with a decreasing trend for the adjusted number of special food services in the 4 surrounding counties (β_31_ = −0.018, *P* = .008) that was coupled with an increasing trend in Philadelphia (β_31_ = 0.017, *P* = .002).

### Drinking places that serve alcoholic beverages

Neither the Philadelphia nor the Pennsylvania CIAA had a significant impact on the trend or level of the adjusted revenue sales for drinking places in any of the 3 regions (Table 1, Figure 1). The Philadelphia CIAA was associated with an increasing trend in the adjusted number of drinking places in the surrounding counties (β_31_ = 0.059, *P* = .02) and the rest of the state (β_31_ = 0.094, *P* = .009) but had no immediate impact (β_21_). The Pennsylvania CIAA was not associated with either the trend or the level of the adjusted number of drinking places in any of the 3 regions (Table 2) (Figure 2).

We noticed seasonal (quarterly) differences in the adjusted sales and adjusted number of food services and drinking places in all 3 regions (Table 1, Table 2, Figure 1, Figure 2). These seasonal differences were found in all 4 types of food services and drinking places: full-service restaurants, limited service eating places, special food services, and drinking places.

## Discussion

Our analysis of the economic impact of indoor smoking bans on Pennsylvania’s eating and drinking establishments did not suggest that businesses in general were harmed. There were 2 instances of immediate impact on food services adjusted taxable revenues, but they were not consistent in their general direction (positive or negative). For the most part, the effect on adjusted taxable sales was small, and the effect on the number of establishments was favorable in comparison to the period before the smoking ban.

Our study was designed to address the predominant limitation of pre-ban and post-ban studies (3,8,12–16), namely the lack of control for confounders and changes in secular trends over time (9,11). Our analytic approach has several unique features compared with other interrupted time-series analyses of economic outcomes in the hospitality industry (19–23). For one, the phased implementation of CIAA statutes in Pennsylvania provided a naturally occurring quasi-experimental study design. The Philadelphia CIAA was implemented approximately 2 years earlier than the Pennsylvania CIAA, and only Philadelphia restaurants and drinking establishments were initially affected. This timing provided an opportunity to evaluate the consistency of the effect of the CIAAs on the food services and drinking places for both CIAAs using the other as a control. Second, in addition to assessing the tax-revenue sales, we also investigated the immediate impact and long-term trend on the number of food services and drinking places. Third, by investigating 4 distinct categories of food service establishments separately, we were able to examine the consistency of findings for different types of food services and drinking places. Few economic studies evaluated food services and drinking places separately. Hence, this assessment adds to the scientific evidence on whether eating and drinking establishments are differentially affected. Fourth, to address the neighborhood effect of the CIAAs on the trend and level of food services and drinking places sales and numbers, analyses were performed for 3 separate regions: Philadelphia, the 4 surrounding counties, and the remainder of the state.

Segmented regression analysis of interrupted time-series was used to account for the trend and autocorrelation. As reported by Glantz and Smith, serial autocorrelation usually exists in time-series data (16). Autocorrelation was also observed in most of our analyses, thus raising concerns that the assumptions of ordinary regression analysis were not met. One key assumption for ordinary regression analysis is that the errors are independent (24). However, with time-series data, the ordinary regression analysis residuals are usually correlated over time; thus, it is appropriate to consider an alternative analytic approach for time-series data. Violation of the independent-errors assumption for ordinary regression analysis could result in erroneous statistical significance of the parameters, and the confidence limits for the predicted value and the estimate of the regression coefficients are not as efficient as they would be if the autocorrelation were taken into account. Our quasi-experimental design, which controls for baseline level and trend when estimating the changes due to CIAAs, addresses the weakness of the simpler pre- and post-proportion comparison methods of ordinary regression that do not consider pre-intervention trends. An evaluation of the rate level and trend before and after the CIAAs is a stronger analytic approach so that an existing trend is not incorrectly interpreted to be related to an intervention, as can occur when performing the ordinary regression analysis.

In addition to the individual models, a combined model with interaction terms was also performed to determine whether the Philadelphia and Pennsylvania CIAA had different effects in the 3 regions. The assumption of this approach was that the trends for the adjusted food services and drinking places sales and numbers among the 3 regions in the state were parallel before the CIAAs. No statistically significant interaction terms were found, indicating that the CIAAs did not have a different effect on the 3 comparison regions.

Our major finding, namely, that indoor smoking bans did not significantly affect quarterly revenue in eating and drinking establishments, is similar to findings in other geographic locations in the United States (3,6,12,25–27) and worldwide (5,7,14,15). Contrary to the lack of relation between Ohio’s ban and economic activity in Kentucky’s border counties (26), the data in Pennsylvania suggest a possible migration of businesses across counties as depicted by the reduction in the number of special food services in the 4 surrounding counties that was coupled with the increase in Philadelphia following the Philadelphia CIAA.

A limitation of our study is that it was not possible to determine which drinking establishments in our analyses were exempt from the CIAA. Thus, the lack of impact of CIAAs may be due to the number of drinking establishments that applied for and received an exemption. The most current 2011 data indicates approximately two-thirds of the drinking places have exemptions (28). Further assessment of exempt versus nonexempt may provide more information. Also, even though we incorporated GMSs as an economic indicator in our analyses of adjusted sales and numbers, there may be some other confounding factors or events, especially those that could affect food services and drinking places differently than the GMSs. These could potentially change the effect of CIAAs on food services and drinking places adjusted sales and numbers.

Our study design addressed the predominant limitation of previous pre-ban and post-ban studies and concerns of opponents of more comprehensive smoking bans about potential loss of revenue. The results of this segmented regression analysis of interrupted time-series approach are consistent with the previous studies and suggest the need to further investigate the impact of CIAAs on drinking places. Overall, the indoor smoking bans had no negative business-related consequences, and for the most part, the data suggested a positive impact. This finding should allay concerns about the economic consequences of smoking restrictions on the hospitality industry and support comprehensive CIAA ordinances to protect the health and well-being of those who patronize and work in these locations, especially in food-service establishments.
